# Modified Shuttle Test Distance Correlates With Peak Oxygen Uptake in Children and Adolescents With Severe Therapy-Resistant Asthma

**DOI:** 10.3389/fphys.2019.01245

**Published:** 2019-10-01

**Authors:** Daniele Schiwe, João Paulo Heinzmann-Filho, Cláudia Silva Schindel, Mailise Fátima Gheller, Natália Evangelista Campos, Paulo Márcio Pitrez, Márcio Vinícius Fagundes Donadio

**Affiliations:** ^1^Laboratório de Atividade Física em Pediatria, Centro Infant, Pontifícia Universidade Católica do Rio Grande do Sul (PUCRS), Porto Alegre, Brazil; ^2^Centro Infant, Pontifícia Universidade Católica do Rio Grande do Sul (PUCRS) e Hospital Moinhos de Vento, Porto Alegre, Brazil

**Keywords:** severe asthma, exercise test, exercise tolerance, physical activity, field tests

## Abstract

**Introduction:**

Several tests may be used to assess exercise intolerance in severe therapy-resistant asthma (STRA), including the gold standard cardiopulmonary exercise test (CPET) and the modified shuttle test (MST).

**Objective:**

To correlate the distance achieved in the MST with peak oxygen uptake (VO_2_peak) and to compare the maximal heart rate (HRmax) obtained in both tests in children and adolescents with STRA.

**Methods:**

This is a cross-sectional study, with 19 children and adolescents with STRA. Demographic, anthropometric, clinical data, and spirometric values were collected. CPET and the MST were performed in two consecutive visits. HRmax, pulse oxygen saturation, and dyspnea were compared between tests. The distance achieved in the MST was correlated with VO_2_peak.

**Results:**

Nineteen patients with a mean age of 11.5 ± 2.5 years were included. The mean HRmax (bpm) achieved was 180.8 ± 12.10 for the MST and 187.6 ± 9.35 for CPET, whereas the mean HRmax as a percentage of predicted (HRmax%) was 90.7 ± 6.5 for the MST and 93.8 ± 4.5 for CPET. A difference of only 6 bpm was found for HRmax (*p* = 0.10) and of 3% for HRmax% (*p* = 0.06) between tests. A strong correlation was found between the MST (*r* = 0.79; *p* = 0.001) and VO_2_peak measured through CPET. However, there were no correlations between the MST and both body mass index (*r* = −0.14; *p* = 0.564) and forced expiratory volume in the first second – FEV_1_ (*r* = −0.02; *p* = 0.917).

**Conclusion:**

The results demonstrate that the MST distance strongly correlates with VO_2_peak, measured through CPET, and the main physiological variable responses were similar between both tests. Our results provide additional data for the use of the MST to assess exercise capacity in children and adolescents with STRA.

## Introduction

Asthma is a chronic respiratory disease characterized by partially and/or fully reversible obstruction of the airways with a high prevalence in the pediatric population (ranging from 2.4 to 37.6%) (The International Study of Asthma and Allergies in Childhood [ISAAC] Steering Committee IS, 1998). Clinically, the signs and symptoms of the disease are the presence of wheezing, dyspnea, chest tightness, and dry cough (Rodrigues et al., 2015). According to data from the World Health Organization (WHO), 300 million people in the world are asthmatics with a worldwide mortality rate of 250,000 persons/year. Among the children with asthma, around 5–10% of the patients have severe asthma (Simões et al., 2010; Rodrigues et al., 2015).

Children with severe therapy-resistant asthma (STRA) are those in which the disease remains uncontrolled, even with optimization of clinical management, or if the disease can only be controlled with GINA step 4 or 5 treatment. These patients present recurrent asthma attacks, with visits to emergencies and frequent hospitalizations, limitation to physical activities, and, consequently, a more sedentary lifestyle. Thus, objective measures of assessment are necessary to determine the exercise capacity of these patients (Carlsen et al., 2011; Chung et al., 2014).

The cardiopulmonary exercise test (CPET) is considered the gold standard test for the evaluation of physical fitness, evaluating the interaction between cardiovascular, respiratory, and metabolic systems, thus providing essential and complex information on exercise limitation. However, although CPET is considered the gold standard test, its execution implies the use of equipment and software with a high cost, limiting its applicability in clinical practice (Ferrazza et al., 2009). Therefore, the evaluation of exercise capacity through simple and low-cost field tests, such as the modified shuttle test (MST), may be an alternative method to measure physical performance in these patients. The MST is an externally paced test with 15 levels and presenting the distance reached in a course of 10 m as its main outcome. Considering that an incremental protocol with increasing speed is used, MST mimics frequently used CPET protocols, leading subjects to levels close to exhaustion (Lanza et al., 2018).

Although several studies (Bradley et al., 1999; Vallier et al., 2016; Lanza et al., 2018; Majd et al., 2019) have shown a good association between maximal oxygen consumption (VO_2_peak) measured during CPET and the distance covered in the MST in adults, little is known for pediatric populations. Evidence suggests differences in both exercise capacity parameters and clinical characteristics of asthma between children and adults (Jenkins et al., 2003; Vinet et al., 2003). To date, only one recent study (Lanza et al., 2018) has evaluated the correlation between these variables in a pediatric sample including mild and moderate asthmatics. Moreover, another study with adult severe asthmatics (Labadessa et al., 2018) showed an important MST reliability. However, there is no evidence on the use of the MST as an exercise capacity assessment tool for children and adolescents with STRA. Thus, the objective of this study was to correlate the distance achieved in the MST with peak oxygen uptake (VO_2_peak) measured through CPET in children and adolescents with STRA. Comparisons of the main physiological responses between tests were also performed.

## Materials and Methods

A cross-sectional study was performed including 19 patients with a confirmed diagnosis of STRA, aged between 6 and 18 years, who were regularly clinically followed at an asthma outpatient clinic. The diagnosis of asthma and the classification of disease severity followed the criteria of the Global Initiative for Asthma (GINA) (Liu et al., 2007) and the American Thoracic Society – European Respiratory Society (ERS–ATS) Guidelines of severe asthma (Chung et al., 2014). Definition of severe asthma for patients aged ≥6 years was: asthma which requires treatment with guidelines suggested medications for GINA steps 4 and 5 asthma (high dose inhaled corticosteroids – ICS and LABA or leukotriene modifier/theophylline) for the previous year or systemic corticosteroids for ≥50% of the previous year to prevent it from becoming “uncontrolled” or which remains “uncontrolled” despite this therapy. Patients with osteoarticular and musculoskeletal abnormalities or unable to perform at least one of the tests were excluded. Children and adolescents were also not recruited to the study during asthma exacerbation but could be later recruited at a time of clinical stability. Clinical stability was defined as the absence of severe exacerbation in the preceding month prior to inclusion, hospital admission within the last 3 months, and/or an admission involving intensive care and intubation within the last year.

The study was approved by the Research Ethics Committee of the University under the number 47845415.4.0000.5336. All parents and/or legal guardians signed the Informed Consent Form and the participants signed an Assent Form. Data collection was performed from March 2016 to April 2018 at the Laboratory of Pediatric Physical Activity (Infant Center – PUCRS).

Parents and/or legal guardians answered the disease control questionnaire (GINA) and after subjects underwent an anthropometric evaluation (weight and height) and a spirometric test. Afterward, the exercise capacity evaluation was performed (MST and CPET). Exercise tests were performed randomly in two consecutive visits (approximately 15 days apart). Both evaluations were performed by three trained researchers with the supervision of one experienced exercise capacity investigator.

### Disease Control

Disease control was assessed using the GINA questionnaire, which is an instrument composed of five questions on symptoms related to the last 4 weeks previous to the interview. This questionnaire provides as final result the categorization of disease control in: controlled asthma (negative answer for all four questions), partially controlled (one to two questions), or uncontrolled (three to four positive answer to questions) (De Onis et al., 2007).

### Anthropometric Data

The anthropometric evaluation was performed by measuring the weight and height in duplicate. The weight measurement was performed in orthostasis, with a minimum of clothing and without footwear, using a digital scale (G-tech, Glass 1 FW, Rio de Janeiro, Brazil) previously calibrated with 100 g precision. The height was obtained through a portable stadiometer (AlturaExata, TBW, São Paulo, Brazil) with a precision of 1 mm, with participants barefoot, feet parallel, ankles joined, and arms extended along the body. From these measurements, the body mass index (BMI) was calculated [weight (kg)/height^2^ (m)] and expressed as absolute values and *z*-score, corrected for age (De Onis et al., 2007).

### Lung Function

Spirometry was performed following the recommendations of the ATS–ERS (Miller et al., 2005). The test was performed with patients in orthostatic position, without the use of a nasal clip, using the KOKO spirometer (Louisville, CO, United States). The spirometric parameters evaluated included forced vital capacity (FVC), forced expiratory volume in the first second (FEV_1_), and forced expiratory flow between 25 and 75% of vital capacity (FEF_25__–__75__%_). Data were expressed as *z*-score based on an international reference equation (Quanjer et al., 2012).

### Cardiopulmonary Exercise Testing

The test was performed following the recommendations of the American Thoracic Society and American College of Chest Physicians (Thompson et al., 2013). The evaluation was performed in a computerized system (Aerograph, AeroSport^®^, United States), coupled to a gas analyzer (VO2000, MedGraphics^®^, United States) using an ergometric treadmill (KT-10400, Inbramed^®^, Brazil). The variables collected during the test included VO_2_peak, carbon dioxide production (VCO_2_), maximal ventilation (V_*E*_), respiratory exchange ratio (RER), ventilatory equivalent for oxygen consumption (V_*E*_/VO_2_) and for carbon dioxide production (V_*E*_/VCO_2_), and maximum heart rate (HRmax). The HRmax was obtained through a HR sensor (Polar H10, Bethpage, NY, United States) directly connected to the gas analysis system, using the highest value that an individual could reach in a maximum effort to the point of exhaustion. The final VO_2_peak results were also normalized using percentiles (Eisenmann et al., 2011).

The test was performed using a ramp protocol adapted from a previous study (Borel et al., 2010). Participants were asked to walk for 2 min to adapt to the treadmill, with a speed of 3 km/h and no inclination. After that, there were increments in the speed of 0.5 km/h every minute, with a fixed slope of 3% until the test was finished (Borel et al., 2010). All patients were encouraged to keep pace until exhaustion or the onset of signs and/or limiting symptoms (dyspnea, leg pain, and/or dizziness). In order to consider the test as maximum, at least three of the following criteria should be observed: exhaustion or inability to maintain the required speed, RER >1.0, HRmax >85% of the HR estimated (208–0.7 × age) and the presence of a plateau for VO_2_ (Rodrigues et al., 2006; Borel et al., 2010). The criteria used to define a plateau was an increase in VO_2_ of <2 mL kg^–1^ min^–1^ during the last minute of test in spite of increasing exercise intensity. At the beginning and the end of the test, data on HR and pulse oxygen saturation (SpO_2_) were collected through a HR sensor (Polar H10, Bethpage, NY, United States) and a pulse oximeter (Nonin^®^, Minneapolis, United States), respectively. Subjective perception of dyspnea and leg fatigue was evaluated using the Modified Borg Scale. HR and SpO_2_ were monitored throughout the CPET protocol.

### Modified Shuttle Test

Modified shuttle test was performed as described by Bradley et al. (1999). Patients were familiarized with the test in a previous appointment. The modified test has 15 levels and patients should walk/run with increasing speeds, in a course of 10 m delimited by two cones that must be circumvented by the patient. An audio signal is an integral part of the test and represents the level change, as well as the increase of the patient’s speed during the test. The test protocol initiated with an average velocity of 0.5 m/s (level 1), followed by an increment of 0.17 m/s at each subsequent level. The patients were followed by a physiotherapist during the first minute in order to adapt to the rhythm of the audio signal. At the beginning of each level, a standardized verbal incentive was offered. The test was completed when participants stated that they were unable to continue the test, lost the rhythm of the audio signal for two consecutive times, or reached the maximum distance of 1500 m.

Before the start of the test and immediately at the end, HR and SpO_2_ (Nonin^®^, Minneapolis, United States) and the modified BORG score for dyspnea and leg fatigue were measured. The distance achieved was calculated by counting the total number of shuttles at the end of the test and expressed in meters. Also, data obtained in the test were normalized using a national reference equation (de Cordoba Lanza et al., 2015).

### Statistical Analysis

The distribution of variables was evaluated using the Shapiro–Wilk test. Symmetric variables were presented as mean and standard deviation and asymmetric variables using median and the interquartile range. Categorical variables were presented in absolute and relative frequency. The delta of the HRmax, HRmax%, and Borg for dyspnea and leg fatigue in both CPET and MST was performed by subtracting peak exercise from resting values. Comparisons between MST and CPET variables were performed with Student’s *t*-test for paired samples. Bland–Altman plots were used to demonstrate HRmax and HRmax% agreement between the MST and CPET. Correlations were assessed using a Pearson linear correlation test. All analyses and data processing were performed in SPSS version 18.0 (SPSS Inc., United States). In all cases, the level of significance adopted was *p* < 0.05.

## Results

Twenty-one patients with STRA were recruited, but two were unable to perform tests adequately. Thus, 19 patients with STRA (63% female), mean age of 11.5 years, were included. Only seven patients (36.8%) presented with controlled asthma. The mean (*z*-score) FEV_1_ was −0.66 and FVC 0.12, demonstrating values within the limits of normality. The characteristics of the sample are presented in Table 1.

**TABLE 1:** Characterization of the study sample.

**Variables evaluated**	**Girls (*n* = 10)**	**Boys (*n* = 9)**	**Total (*n* = 19)**
**Demographic characteristics**			
Age (years)	12.2 ± 2.5	10.6 ± 1.8	11.5 ± 2.5
**Anthropometry**			
Height (cm)	145.3 ± 12.2	144.1 ± 11.9	144.7 ± 12.5
Weight (kg)	45.0 ± 10.4	44.4 ± 14.0	44.7 ± 12.6
BMI (absolute)	21.1 ± 3.7	21.5 ± 7.8	21.3 ± 6.2
(*z*-score)	0.6 ± 1.0	0.7 ± 1.0	0.6 ± 1.1
**Disease control, *n* (%)**			
Controlled	1 (10.0)	5 (55.5)	6 (31.6)
Partially controlled	5 (50.0)	1 (11.1)	6 (31.6)
Uncontrolled	4 (40.0)	3 (33.3)	7 (36.8)
**Lung function**			
FEV_1_ (absolute)	2.2 ± 0.5	1.8 ± 0.4	2.0 ± 0.5
(*z*-score)	−0.1 ± 1.7	−1.3 ± 1.3	−0.6 ± 1.7
FVC (absolute)	2.6 ± 0.6	2.5 ± 0.5	2.6 ± 0.6
(*z*-score)	0.3 ± 1.4	−0.1 ± 1.0	0.1 ± 1.3
VEF_1_/CVF (absolute)	0.8 ± 0.0	0.7 ± 0.1	0.8 ± 0.1
FEF_25__–__75__%_ (absolute)	2.7 ± 1.2	1.6 ± 0.7	2.2 ± 1.2
(*z*-score)	−0.3 ± 1.8	−1.9 ± 1.5	−1.1 ± 1.9

*BMI, body mass index; FEV_1_, forced expiratory volume in 1 s; FVC, forced vital capacity; FEF_25__–__75__%_, forced expiratory flow between 25 and 75% of vital capacity. Values expressed as mean ± standard deviation, except for the control variables of the disease and sex (absolute and relative frequency).*

The mean level achieved during the MST was 11.1 ± 1.7 and the average distance reached was 693.8 ± 293.3 m (92.2% of predicted). Regarding variables evaluated on CPET, the mean VO_2_peak was 34.6 ± 7.9 mL kg^–1^ min^–1^, V_*E*_ was 39.6 ± 11.5 L min^–1^ and the median VO_2_peak was found at the 25th percentile (3.5–50). The mean HRmax (bpm) achieved was 180.8 ± 12.1 for the MST and 187.6 ± 9.3 for CPET, whereas the mean HRmax as a percentage of predicted (HRmax%) was 90.7 ± 6.5 for the MST and 93.8 ± 4.5 for CPET. A mean difference of only 6 bpm was found for HRmax (*p* = 0.06) and of 3% for HRmax% (*p* = 0.08) between the tests. A significant decrease in the final SpO_2_ and Borg for dyspnea was found when CPET was compared to the MST (Table 2).

**TABLE 2:** Resting characteristics and physiological responses to exercise testing.

**Variables evaluated**	**MST**	**CPET**	***p*-value**
**Resting**			
Heart rate (beats/min)	87.9 ± 16.1	89.4 ± 13.6	0.53
SpO_2_ (%)	98.3 ± 0.83	98.1 ± 1.5	0.49
Borg dyspnea	0.0 (0.0–0.0)	0.0 (0.0–0.2)	0.59
Borg leg fatigue	0.0 (0.0–0.5)	0.0 (0.0–1.0)	0.29
**Physiological responses**			
MST level	11.1 ± 1.7	–	–
MST distance (m)	693.8 ± 293.3	–	–
MST (%)	92.2 ± 19.2	–	
VO_2_ peak (mL kg^–1^ min^–1^)	–	34.6 ± 7.9	–
VO_2_ percentile^#^	–	25 (3.5–50)	–
V_E_ peak (L min^–1^)	–	39.6 ± 11.5	–
V_E_/VO_2_ peak (L min^–1^)	–	23.5 ± 1.7	–
V_E_/VCO_2_ peak (L min^–1^)	–	23.2 ± 1.7	–
RER	–	1.1 ± 0.06	–
Max heart rate (beats min^–1^)	180.8 ± 12.1	187.6 ± 9.3	0.06
Max heart rate (% predicted)	90.7 ± 6.5	93.8 ± 4.5	0.08
End SpO_2_ (%)	98.0 ± 1.4	95.6 ± 2.6	<0.01^∗^
End Borg dyspnea	4.0 (2.0–5.0)	2.0 (1.0–4.0)	0.01^∗^
End Borg leg fatigue	2.0 (1.0–5.0)	3.0 (2.0–5.0)	0.62

*MST, modified shuttle test; CPET, cardiopulmonary exercise testing; SpO_2_, pulse oxygen saturation; VO_2_, maximal oxygen uptake; V_*E*_, minute ventilation; V_*E*_/VO_2_, ventilatory equivalent ratio for oxygen uptake; V_*E*_/VCO_2_, ventilatory equivalent ratio for carbon dioxide production; RER, respiratory exchange ratio. Values expressed as mean ± standard deviation and # median and interquartile range. ^∗^indicate significant differences (*p* < 0.05).*

**FIGURE 1:**
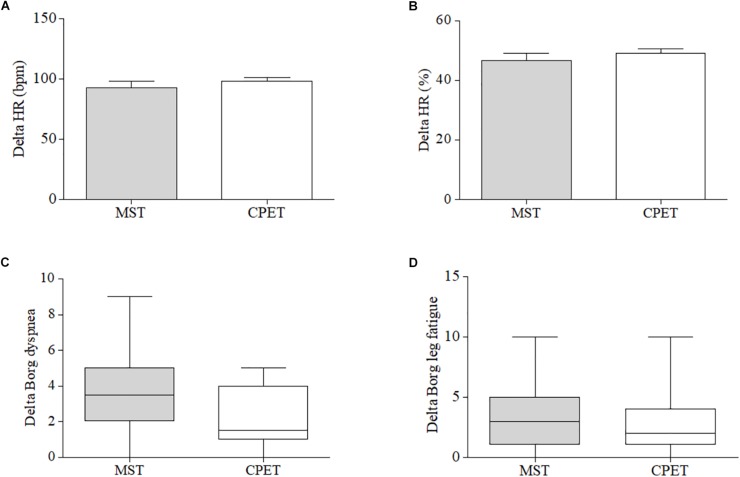
Comparison of the difference (delta) between peak and resting values (bpm) of **(A)** maximum heart rate (HRmax), **(B)** percentage of HRmax and subjective perception of **(C)** dyspnea and **(D)** leg fatigue.

**FIGURE 2:**
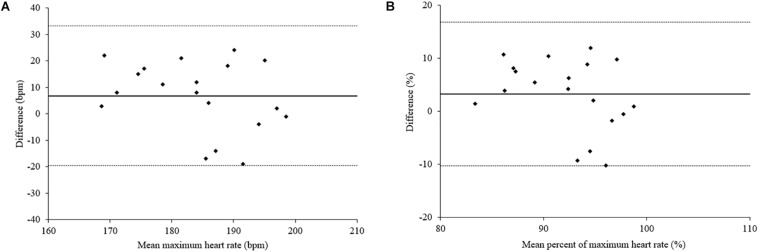
Bland–Altman plot showing individual differences between **(A)** maximum heart rate (HRmax) and **(B)** percent of maximum heart rate (HRmax%) during CPET *versus* the MST. The solid line indicates the mean difference between paired measurements and the dotted lines indicate the 95% limits of agreement.

We have also evaluated differences between peak exercise and resting values (delta) for both tests and no significant differences were found for HRmax, HRmax%, Borg for dyspnea, and Borg for leg fatigue, when MST and CPET were compared (Figure 1). In addition, the Bland–Altman plot shows the agreement of HRmax and HRmax% between the MST and CPET (Figure 2).

**FIGURE 3:**
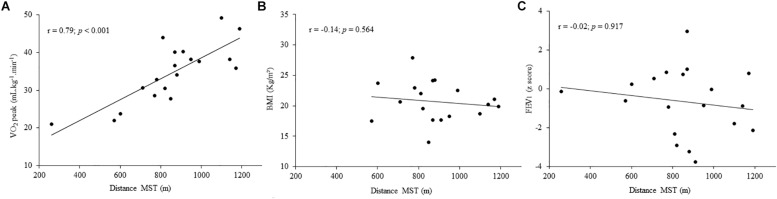
Correlation between the distance achieved in the modified shuttle test (MST) and **(A)** peak oxygen consumption (VO_2_peak) measured during cardiopulmonary exercise testing (CPET), **(B)** body mass index (BMI), and **(C)** forced expiratory volume in the first second (FEV1).

A strong significant correlation (*r* = 0.79; *p* = 0.001) was found between VO_2_peak and the distance achieved in the MST. However, there were no correlations between the MST and both BMI (*r* = −0.14; *p* = 0.564) and FEV_1_ (*r* = −0.02; *p* = 0.917) (Figure 3).

## Discussion

The results of the present study demonstrated that the MST distance correlates strongly with maximal oxygen uptake measured through CPET in children and adolescents with STRA. In addition, a similar response between tests was demonstrated for HRmax. We believe this is the first study to use the MST for the evaluation of exercise capacity in pediatric STRA patients. Considering that the test is relatively easy-to-perform, safe, and showed an important correlation with CPET, it may be considered as an alternative to evaluate exercise capacity in this population.

Although the benefits of physical exercise are well known, including positive cardiovascular and musculoskeletal effects, quality of life improvement, and better disease control (Coelho et al., 2018), some studies (Pianosi and Davis, 2004; Coelho et al., 2018; Furtado et al., 2018) demonstrate that adult patients with STRA present significant impairment of physical activity levels. In our study, VO_2_ was reduced when evaluated in both CPET [six patients (31.6%) with the percentile for VO_2_ < 5] and MST [six patients (31.6%) showing values < 80% of predicted], suggesting a compromised exercise capacity. The reduction of physical performance in asthmatics could be attributed to the degree of airway obstruction, the occurrence of exercise-induced bronchoconstriction (EIB), decreased respiratory capacity, and a greater sensation of dyspnea (Costa et al., 2018). Thus, the early evaluation of exercise capacity is important, showing an objective information to the patient in order to indicate aerobic training programs, as it is already known that inactive youths tend to become sedentary adults (Leinaar et al., 2016).

Cardiopulmonary exercise test is considered the gold standard for assessing physical fitness. However, this method requires costly equipment, specialized training, and is not always available in clinical practice (Society, 2003), especially in developing countries. Thus, it is often necessary to determine exercise capacity using field tests and the MST is considered a simple, externally paced, and incremental (progressive increase of velocity) test (Lanza et al., 2018). Several studies (Costa et al., 2018; Labadessa et al., 2018; Lanza et al., 2018; Vendrusculo et al., 2019) have demonstrated good correlations between the distance achieved in the MST and the VO_2_ obtained during CPET, both in respiratory diseases and in other clinical conditions. Recently, one study (Lanza et al., 2018) showed a strong correlation between the MST and VO_2_ (87% prediction power) in children and adolescents with mild/moderate asthma. These results are similar to ours, considering that we have demonstrated a strong correlation between the MST distance and the VO_2_peak measured on CPET. To the best of our knowledge, this is the first study to correlate the distance achieved in the MST with VO_2_peak in children and adolescents with STRA. Our results corroborate previous research showing a good agreement between CPET and the MST, both in pediatric samples (Lanza et al., 2018) and in the adult population (Costa et al., 2018; Labadessa et al., 2018).

A previous study from our group (de Assumpção et al., 2018) showed a negative correlation between nutritional status and MST distance in obese children and adolescents with no respiratory disease. Increased nutritional status leads to an extra body demand to be transferred during daily tasks and to the tendency of subjects to become even more inactive (Bovet et al., 2007; Castro-Piñero et al., 2011). However, present results demonstrated no correlation between BMI and distance achieved in the MST. Although high BMI is commonly seen in adults with asthma (Costa et al., 2018; Majd et al., 2019) and may influence exercise capacity, only one previous study demonstrated the deleterious influence of nutritional status on physical performance in asthmatic children (Pianosi and Davis, 2004). In addition, the lung function showed no correlations with the MST. It is possible that this finding is related to the fact that most patients presented normal lung function.

Although HRmax was slightly higher on CPET, no significant differences were found, as well as mean values of HRmax obtained in both tests were >180 bpm, indicating a maximum effort level for both tests. These results are in agreement with the study of Gelbart et al. (2017), which suggests using 180 bpm as a cut-off value for HRmax in children and adolescents. Also, the findings of the present study confirm a similar HR physiological response between the MST and CPET for children and adolescents with STRA. The mean HRmax difference between tests was only 6 bpm, below that found (13 and 16 bpm) by other studies (Vallier et al., 2016; Lanza et al., 2018). Regarding SpO_2_, lower values were measured on CPET compared to the MST. However, as values are within a range of normality, we believe this finding is not clinically relevant. As for the subjective sensation of dyspnea (Borg), there is evidence (Vallier et al., 2016) showing a greater sensation in the MST compared to CPET, which is in agreement with our results. It is also interesting to note that low scores of dyspnea were found at peak for both tests. Although there are no current explanations available, studies (Lanza et al., 2018; Majd et al., 2019) have also reported low levels of dyspnea evaluated through the Borg scale during maximum tests in asthmatic patients. Nevertheless, these differences should be interpreted with caution, considering that the Borg subjective scale does not present adequate sensitivity for children under the age of 9 years (Hommerding et al., 2010).

Finally, the small number of patients and the impossibility of directly measuring gases during the MST were limitations of our study. Portable expiratory gas analysis was not performed during the MST to assess whether there was the same linear increase in oxygen uptake and VO_2_peak as achieved in CPET. However, our study showed that there was a strong correlation between the MST distance and VO_2_peak on CPET, as well as in clinical practice the outcome measure for the MST is the distance covered by the individual. In addition, given that STRA in children and adolescents is an uncommon clinical phenotype (around 1% of asthmatics), our sample size and power seems adequate for this type of study (Costa et al., 2018; Labadessa et al., 2018). In addition, although a maximal response was obtained, it is possible that learning effects may have influenced results, as only one MST was performed.

## Conclusion

In conclusion, the results of our study demonstrate that the distance achieved in the MST correlates with VO_2_peak measured through CPET in children and adolescents with STRA. In addition, the main physiological variable responses were similar between both tests. Our results provide additional data for the use of the MST to assess exercise capacity in children and adolescents with STRA.

## Data Availability Statement

The datasets generated for this study are available on request to the corresponding author.

## Ethics Statement

The study was approved by the Research Ethics Committee of the University under the number 47845415.4.0000.5336. All parents and/or legal guardians signed the Informed Consent Form and the participants signed an Assent Form.

## Author Contributions

MD had substantial contributions to the study including conceptualization and design, supervision and oversight, formal analysis, and drafting significant parts of the work. DS, JH-F, and PP had substantial contributions to the study including analysis and interpretation of research data, and drafting significant parts of the work or critically revising. DS, CS, MG, and NC had substantial contributions to the study including methodology, data collection, data curation, and critically revising.

## Conflict of Interest

The authors declare that the research was conducted in the absence of any commercial or financial relationships that could be construed as a potential conflict of interest.

## References

[B1] Borel B. Leclair E. Thevenet D. Beghin L. Berthoin S. Fabre C. (2010). Correspondences between continuous and intermittent exercises intensities in healthy prepubescent children. *Eur. J. Appl. Physiol.* 108 977–985. 10.1007/s00421-009-1296-y 19960352

[B2] Bovet P. Auguste R. Burdette H. (2007). Strong inverse association between physical fitness and overweight in adolescents: a large school-based survey. *Int. J. Behav. Nutr. Phys. Act.* 4:24. 10.1186/1479-5868-4-24 17550617 PMC1894813

[B3] Bradley J. Howard J. Wallace E. Elborn S. (1999). Validity of a modified shuttle test in adult cystic fibrosis. *Thorax* 54 437–439. 10.1136/thx.54.5.437 10212110 PMC1763768

[B4] Carlsen K. L. Hedlin G. Bush A. Wennergren G. de Benedictis F. M. De Jongste J. (2011). Assessment of problematic severe asthma in children. *Eur. Respir. Soc.* 37 432–440. 10.1183/09031936.00091410 21030450

[B5] Castro-Piñero J. Ortega F. Keating X. D. González-Montesinos J. Sjöstrom M. Ruíz J. R. (2011). Percentile values for aerobic performance running/walking field tests in children aged 6 to 17 years influence of weight status. *Nutr. Hosp.* 26 572–578. 10.1590/S0212-16112011000300021 21892577

[B6] Chung K. F. Wenzel S. E. Brozek J. L. Bush A. Castro M. Sterk P. J. (2014). International ERS/ATS guidelines on definition, evaluation and treatment of severe asthma. *Eur. Respir. J.* 43 343–373. 24337046 10.1183/09031936.00202013

[B7] Coelho C. M. Reboredo M. M. Valle F. M. Malaguti C. Campos L. A. Nascimento L. M. (2018). Effects of an unsupervised pedometer-based physical activity program on daily steps of adults with moderate to severe asthma: a randomized controlled trial. *J. Sports Sci.* 36 1186–1193. 10.1080/02640414.2017.1364402 28799458

[B8] Costa I. P. Dal Corso S. Borghi-Silva A. Peixoto F. Stirbulov R. Arena R. (2018). Reliability of the shuttle walk test with controlled incremental velocity in patients with difficult-to-control asthma. *J. Cardiopulm. Rehabil. Prev.* 38 54–57. 10.1097/HCR.0000000000000286 28885280

[B9] de Assumpção P. K. Heinzmann-Filho J. P. Isaia H. A. Holzschuh F. Dalcul T. Donadio M. V. F. (2018). Exercise capacity assessment by the modified shuttle walk test and its correlation with biochemical parameters in obese children and adolescents. *Indian J. Pediatr.* 85 1079–1085. 10.1007/s12098-018-2649-5 29569079

[B10] de Cordoba Lanza F. do Prado Zagatto E. Silva J. C. Selman J. P. R. Imperatori T. B. G. Zanatta D. J. M. (2015). Reference equation for the incremental shuttle walk test in children and adolescents. *J. Pediatr.* 167 1057–1061. 10.1016/j.jpeds.2015.07.068 26323195

[B11] De Onis M. Garza C. Onyango A. W. Borghi E. (2007). Comparison of the WHO child growth standards and the CDC 2000 growth charts. *J. Nutr.* 137 144–148. 10.1093/jn/137.1.144 17182816

[B12] Eisenmann J. C. Laurson K. R. Welk G. J. (2011). Aerobic fitness percentiles for US adolescents. *Am. J. Prev. Med.* 41 S106–S110. 21961609 10.1016/j.amepre.2011.07.005

[B13] Ferrazza A. Martolini D. Valli G. Palange P. (2009). Cardiopulmonary exercise testing in the functional and prognostic evaluation of patients with pulmonary diseases. *Respiration* 77 3–17. 10.1159/000186694 19145106

[B14] Furtado P. R. Maciel ÁC. C. Barbosa R. R. T. da Silva A. A. M. de Freitas D. A. de Mendonça K. M. P. P. (2018). Association between quality of life, severity of asthma, sleep disorders and exercise capacity in children with asthma: a cross-sectional study. *Braz. J. Phys. Ther.* 23 12–18. 10.1016/j.bjpt.2018.08.010 30166089 PMC6546840

[B15] Gelbart M. Ziv-Baran T. Williams C. A. Yarom Y. Dubnov-Raz G. (2017). Prediction of maximal heart rate in children and adolescents. *Clin. J. Sport Med.* 27 139–144. 10.1097/jsm.0000000000000315 27177205

[B16] Hommerding P. X. Donadio M. V. Paim T. F. Marostica P. J. (2010). The borg scale is accurate in children and adolescents older than 9 years with cystic fibrosis. *Respir. Care* 55 729–733. 20507656

[B17] Jenkins H. A. Cherniack R. Szefler S. J. Covar R. Gelfand E. W. Spahn J. D. (2003). A comparison of the clinical characteristics of children and adults with severe asthma. *Chest* 124 1318–1324. 10.1378/chest.124.4.1318 14555561

[B18] Labadessa I. G. Borghi-Silva A. de Araujo A. S. G. Rizzatti F. P. G. Di Lorenzo V. A. P. (2018). Reliability of cardiorespiratory and metabolic responses during incremental shuttle walk test in adult subjects with asthma. *Respir. Care* 64 55–62. 10.4187/respcare.06112 30154128

[B19] Lanza F. C. Reimberg M. M. Ritti-Dias R. Scalco R. S. Wandalsen G. F. Sole D. (2018). Validation of the modified shuttle test to predict peak oxygen uptake in youth asthma patients under regular treatment. *Front. Physiol.* 9:919. 10.3389/fphys.2018.00919 30087618 PMC6066955

[B20] Leinaar E. Alamian A. Wang L. (2016). A systematic review of the relationship between asthma, overweight, and the effects of physical activity in youth. *Ann. Epidemiol.* 26 504–510.e6. 10.1016/j.annepidem.2016.06.002 27449571

[B21] Liu A. H. Zeiger R. Sorkness C. Mahr T. Ostrom N. Burgess S. (2007). Development and cross-sectional validation of the childhood asthma control test. *J. Allergy Clin. Immunol.* 119 817–825. 10.1016/j.jaci.2006.12.662 17353040

[B22] Majd S. Hewitt S. M. Apps L. D. Murphy A. C. Bradding P. Singh S. J. (2019). Understanding the measurement properties of the incremental shuttle walk test in patients with severe asthma. *Respirology* 24 752–757. 10.1111/resp.13519 30887627

[B23] Miller M. R. Hankinson J. Brusasco V. Burgos F. Casaburi R. Coates A. (2005). Standardisation of spirometry. *Eur. Respir. J.* 26 319–338. 10.1183/09031936.05.00034805 16055882

[B24] Pianosi P. T. Davis H. S. (2004). Determinants of physical fitness in children with asthma. *Pediatrics* 113:e225-9. 14993581 10.1542/peds.113.3.e225

[B25] Quanjer P. H. Stanojevic S. Cole T. J. Baur X. Hall G. L. Culver B. (2012). Multi-ethnic reference values for spirometry for the 3-95 year age range: the global lung function equations. *Eur. Respir. J.* 40 1324–1343. 10.1183/09031936.00080312 22743675 PMC3786581

[B26] Rodrigues A. M. Roncada C. Santos G. Heinzmann-Filho J. P. Souza R. G. D. Vargas M. H. M. (2015). Clinical characteristics of children and adolescents with severe therapy-resistant asthma in Brazil. *J. Bras. Pneumol.* 41 343–350. 10.1590/s1806-37132015000004462 26398754 PMC4635954

[B27] Rodrigues A. N. Perez A. J. Carletti L. Bissoli N. S. Abreu G. R. (2006). Maximum oxygen uptake in adolescents as measured by cardiopulmonary exercise testing: a classification proposal. *J. de Pediatr.* 82 426–430. 10.2223/JPED.1533 17003945

[B28] Simões S. D. M. Cunha S. S. D. Barreto M. L. Cruz ÁA. (2010). Distribution of severity of asthma in childhood. *J. de Pediatr.* 86 417–423. 10.2223/JPED.2030 20938593

[B29] Society A. T. (2003). ATS/ACCP statement on cardiopulmonary exercise testing. *Am. Respir. Crit. Care Med.* 167:211. 10.1164/rccm.167.2.211 12524257

[B30] The International Study of Asthma and Allergies in Childhood [ISAAC] Steering Committee IS (1998). Worldwide variations in the prevalence of asthma symptoms: ISAAC. *Eur. Respir. J.* 12 315–335. 10.1183/09031936.98.12020315 9727780

[B31] Thompson P. D. Arena R. Riebe D. Pescatello L. S. (2013). ACSM’s new preparticipation health screening recommendations from ACSM’s guidelines for exercise testing and prescription. *Curr. Sports Med. Rep.* 12 215–217. 10.1249/jsr.0b013e31829a68cf 23851406

[B32] Vallier J.-M. Rouissi M. Mely L. Gruet M. (2016). Physiological responses of the modified shuttle test in adults with cystic fibrosis. *J. Cardiopulm. Rehabil. Prev.* 36 288–292. 10.1097/HCR.0000000000000181 27182761

[B33] Vendrusculo F. M. Heinzmann-Filho J. P. Campos N. E. Gheller M. F. de Almeida I. S. Donadio M. V. (2019). Prediction of peak oxygen uptake using the modified shuttle test in children and adolescents with cystic fibrosis. *Pediatr. Pulmonol.* 54 386–392. 10.1002/ppul.24237 30614221

[B34] Vinet A. Mandigout S. Nottin S. Nguyen L. Lecoq A.-M. Courteix D. (2003). Influence of body composition, hemoglobin concentration, and cardiac size and function of gender differences in maximal oxygen uptake in prepubertal children. *Chest* 124 1494–1499. 10.1378/chest.124.4.1494 14555585

